# Effect of Colloidal Silica on the Hydration Behavior of Calcium Aluminate Cement

**DOI:** 10.3390/ma11101849

**Published:** 2018-09-28

**Authors:** Feng Wang, Pingan Chen, Xiangcheng Li, Boquan Zhu

**Affiliations:** The State Key Laboratory of Refractories and Metallurgy, Wuhan University of Science and Technology, Wuhan 430081, China; infinityqd@126.com (F.W.); zbqref@263.net (B.Z.)

**Keywords:** colloidal silica, calcium aluminate cement, hydration

## Abstract

The effect of colloidal silica (CS) on the hydrate phases and microstructure evolution of calcium aluminate cement (CAC) was investigated. Samples hydrated with CS were obtained and characterized by X-ray diffraction (XRD), Field Emission Scanning Electron Microscopy (FESEM), Fourier Transform Infrared spectroscopy (FT-IR), hydration heat measurement and Nuclear Magnetic Resonance (NMR). The results revealed that SiO_2_ nanoparticles may affect the hydrates crystallization process. There was a compact structure in the CAC paste with CS, while petal-shaped hydrates with a porous structure were formed in the pure CAC paste. The maximum value of electrical conductivity for CAC paste with CS suggested that the early stage of hydration for CAC was accelerated. However, the hydration heat curves revealed that the late stage of the CAC hydration process was inhibited, and the hydration degree was reduced, this result was in accordance with Thermogravimetry-Differential scanning calorimetry(TG-DSC) curves. The fitting results of hydration heat curves further showed that the hydration degree at NG (nucleation and crystal growth) process stage was promoted, while it was limited at the phase boundaries stage, and the diffusion stage in the hydration reaction was brought forward due to the addition of CS. According to these results and analyses, the differences in the hydration process for CAC with and without CS can be attributed to the distribution and nucleation effect of SiO_2_ nanoparticles.

## 1. Introduction

Calcium aluminate cement (CAC) has been widely applied in many industrial applications owing to its advanced properties, such as suitable rheological properties for workability in refractory castables, outstanding green mechanical strength and resistance to a wide range of chemically aggressive conditions [1,2,3,4]. Nevertheless, one of the disadvantages related to its use for cement-based products in industrial applications is its low strength during the curing and dewatering steps, which must be carefully conducted to reduce the possibility of explosive spalling [5,6,7,8].

In recent years, nano-bonded products have been presented as an alternative to overcome the above problems [9,10,11,12]. Further, using nano-powders and colloidal suspensions to improve the bonding strength and densification during the curing stage has attracted great interest. The setting of nano-bonded products is induced by gelation of the nanoparticles [11]. Taking colloidal silica (CS) as an example, the hydroxyl groups (Si-OH) on the surface of the colloidal particles generate siloxane bonds (Si-O-Si) during setting, giving rise to a three-dimensional network [13,14]. Despite the advantages of colloidal silica, there are still some problems hindering its potential applications. The available setting times are long and the as-cast green mechanical strength is lower than that of CAC-bonded products [13,15]. Ismael et al. [13] found that a colloidal silica-bonded composition cured at 50 °C in a moisture saturated environment did not obtain enough mechanical strength even after 168 h. To overcome the problem, Wang et al. [9] used a silane coupling agent to accelerate the setting process of colloidal silica to improve its green strength. Khezrabad et al. [11] used CAC as a gelling agent and obtained high green strength of colloidal silica bonded castables.

According to the features of CAC and CS, the use of CAC cement or colloidal silica alone cannot meet industry requirements for high performance castables. Thus, in order to use their advantages and avoid their shortcomings, the combination of CAC cement and colloidal silica is expected to obtain excellent green mechanical strength and bonding strength at elevated temperatures. However, little attention has been paid to the effect of silica nanoparticles on the hydration properties of CAC cement. Though the main hydration processes are similar for Portland cement and CAC cement, there are differences in the specific hydration processes. For example, Portland cement paste is enriched with Ca^2+^, OH^−^, SO_4_^2−^ and silicate ions, and the solution becomes supersaturated with respect to the precipitation of calcium-silicate-hydrate (C-S-H). However, Ca^2+^ and Al(OH)_4_^−^ ions are dissolved in CAC cement as cement is mixed in the water. After the dissolution process, the hydrates are precipitated to produce CaO⋅Al_2_O_3_⋅10H_2_O (CAH_10_), 2CaO⋅Al_2_O_3_⋅8H_2_O (C_2_AH_8_), 3CaO⋅Al_2_O_3_⋅6H_2_O (C_3_AH_6_) and Al_2_O_3_⋅3H_2_O (AH_3_). The C-S-H in the Portland cement is substituted by hydrated calcium aluminate in the CAC paste, which is responsible for the high green strength of CAC-bonded products. However, CAH_10_ and C_2_AH_8_ are transformed to C_3_AH_6_ and AH_3_ as the hydration process proceeds, and these hydrates are dehydrated to CA and CA_2_ as the temperature increases. Both the transformation and dehydration processes lead to the strength drastically decreasing for CAC bonded products [16,17]. Therefore, colloidal silica may greatly influence the CAC cement hydration process and phases.

Mostafa [1] found that the addition of silica fume to CAC cement prevented the strength loss of castables because the conversion reactions from CAH_10_ to C_2_AH_8_ and C_3_AH_6_ were prevented, and Son [18] found similar results using nano-silica as an additive in CAC cement. Midgley et al. [19] reported that micro-silica enabled the formation of strätlingite, which showed better mechanical properties than hydrogarnet (C_3_AH_6_). Xi et al. [20] found that silica fume favored the formation of a dense pore structure in CAC cement paste. Jime´nez [21] investigated the effect of sodium silicate on CAC hydration in alkaline media and found that the high alkali content expedited the conversion from metastable hydrates to stable hydrates. Though some research has been carried out on the effect of silica fume or nano-silica on hydration behavior, the influence of colloidal silica on hydration behavior is not clear, especially as systematical research on the hydration mechanism of CAC cement with colloidal silica is little reported. 

Thus, CAC cement hydration characteristics in the presence of colloidal silica at the early and late ages were systematically investigated in this study, including the evolution of the phases and morphologies of hydrates, FT-IR spectrums of hydrates, electrical conductivity, hydration heat evolution and hydration kinetics. All these results could provide a comprehensive explanation for the effect of colloidal silica on the hydration process of CAC cement. 

## 2. Experimental

The raw materials used in this work were calcium aluminate cement (CAC), colloidal silica (CS) and polycarboxylate superplasticizer. The CAC cement and CS were provided by Almatis Aluminum (Qingdao) Co., Ltd., Qingdao, China and Hubei Jinwei Group Co Ltd., Wuhan, China respectively. The CS was produced using the sol-gel technique and stabilized by sodium; the average particle size of the SiO_2_ nanoparticles was 5–10 nm. The superplasticizer based on polycarboxylate superplasticizer is an opaque light yellow liquid, with a density of 1.08 g/mL. The chemical analysis of the CAC cement and CS is given in Table 1, the chemical compositions in which were supplied by the relevant companies.

**Figure 1:**
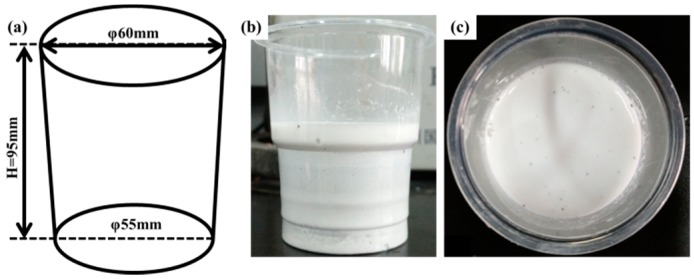
The dimensions of hydrated sample. (**a**) The dimension of plastic cup; (**b**,**c**) the hydrated sample.

In the experiment, the CS and superplasticizer were added by the weight of CAC. The proportion of added CS was increased from 0 to 100 wt.% (0, 20, 60, 100 wt.%, as shown in Table 2), while the weight of the cement remained the same. The 0.2 wt.% superplasticizer (total weight) was mixed with CAC cement. Then CAC cement and CS were mixed with a constant water-cement (pure CAC cement) ratio (w/c = 0.4) for 1 min. After that, the pastes were transferred into plastic cup and the cups were kept in a thermostatic cabinet (HH-1, Changzhou Aohua Instrument Co., Ltd., Changzhou, China) at 100% relative humidity for 12 h, 24 h and 48 h at 30 °C. The plastic cup was cone, its dimension was shown in Figure 1a. The hydrated sample was shown in Figure 1b,c.

After the pastes were hydrated for the specified time, the freeze drying method was carried out to stop the hydration process. The specific method was to put the pastes into the refrigerator (BCD-213WTM(E), Midea Group Co., Ltd., Foshan, China) for 24 h, so the free water became ice. After that, the pastes were put into a freeze dryer for 24 h to remove any remaining free water. The obtained samples were ground into powders for XRD, FTIR and NMR tests. The fracture morphologies were performed by FESEM using the broken samples. The phase compositions of hydrates were evaluated by X-ray diffraction (XRD, X’Pert Pro, Philips, Amsterdam, Netherlands), with a copper anode (Cu K_α_ λ = 0.15405 nm). Scans were performed in the continuous mode at a 2°/min scan rate. The experimental diffraction patterns were compared with International Center for Diffraction Data (ICDD) files. The microstructures of hydrates were characterized by field-emission scanning electron microscopy (FESEM, Nova 400 Nano SEM, FEI, ThermoFisher Scientific Company, Hillsboro, AL, USA) and energy-dispersive spectroscopy (EDS, INCA, IE350 penta FET X-3, Oxford Instruments, Oxford, UK). Fourier transform infrared spectrum (FT-IR) spectroscopic analysis (Nexus, Thermo Nicolet 470, Perkin-Elmer Co., Ltd., Waltham, MA, USA) was carried out to investigate the existence of chemical bonds in the hydrates. The FT-IR spectral analysis was recorded from 400 cm^−1^ up to 4000 cm^−1^. Solid state NMR spectra were acquired on a Bruker Avance III HD 400 MHz (Bruker Co., Ltd., Bill ricard, MA, USA) spectrometer equipped with a wide bore 9.4 Tesla magnet for ^29^Si. All NMR spectra were fitted with DMFIT functions for quantitative deconvolution of overlapping peaks. 

The hydration heat was measured at 30 °C by micro calorimeter (Setaram C80, SETARAM Instrumentation Co., Ltd., Caluire, France) and the water-cement ratio (w/c) was 1. The electrical conductivity of the pastes (w/c = 2.3) was measured by mixing CAC cement and CS. The specific measurement conditions of electrical conductivity were as follows. The CAC cement and CS were mixed for 1 min continuously. After that, pastes were transferred into a cylindrical plastic sample holder (20 mm internal diameter), and glass electrodes was protruded into the pastes and the cell was kept in a thermostatic cabinet at 100% relative humidity during the test period. The measurement began after the pastes were mixed for 2 min and the measurement lasted for 72 h at 30 °C. Thermal characterizations were conducted using differential scanning calorimetry and thermogravimetric analysis (STA449C/3/g, NETZSCH, Seklb, Germany) with a heating rate of 10 °C/min up to 800 °C under air flow. 

## 3. Results and Discussion

### 3.1. Phase and Microstructure Evolution of Hydrates

**Figure 2:**
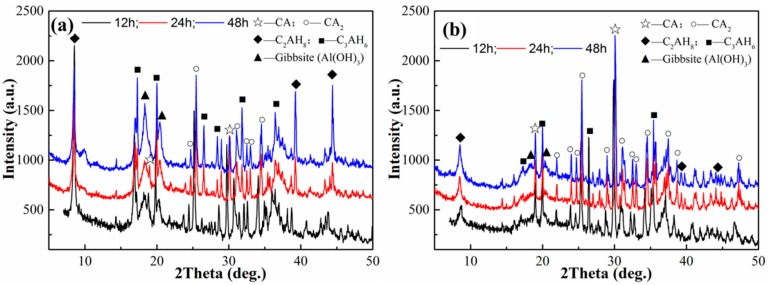
XRD patterns of cement paste after different hydration times: (**a**) CAC; (**b**) CAC with 100 wt.% CS.

Figure 2 shows the XRD patterns of the CAC paste without and with 100 wt.% CS after different hydration times. It can be seen from Figure 2a,b that the diffraction peaks of hydrates such as C_2_AH_8_, C_3_AH_6_ and gibbsite are obvious and intensities of these peaks are increased with hydration time increases. In Figure 2b, the hydrate tapes are the same as those in Figure 2a, and the same trend of hydrate peak intensities can be seen with hydration time increases. However, when the hydration time is equal, the intensities of C_2_AH_8_ and C_3_AH_6_ peaks in Figure 2b are weaker than those in Figure 2a, while the intensities of CA and CA_2_ peaks are higher in Figure 2b.

The hydration process begins with the dissolution of the anhydrous phase and is followed by the precipitation of thermodynamically stable hydrates. Three distinct steps can be identified; dissolution, nucleation and precipitation [22]. The hydration process is initiated by the hydroxylation of calcium aluminate and followed by the dissolution of Ca in water and the formation of Ca^2+^ and Al(OH)_4_^−^ species. Hydrates such as CAH_10_, C_2_AH_8_ and C_3_AH_6_ are precipitated as the solution concentration surpasses the solubility. Therefore, the differences of hydrate XRD patterns in Figure 2 indicate that the SiO_2_ nanoparticles affect the formation of crystalline hydrates, such as C_2_AH_8_ and C_3_AH_6_. The low crystallization of CAC hydrates resulting from the colloidal silica is also consistent with reference [18]. It is noted that Ca_2_Al_2_SiO_7_·8H_2_O (C_2_ASH_8_) is not detected in the CAC paste with CS. This may be due to the poor crystallization of C_2_ASH_8_. Similar results have been found in other research [23].

**Figure 3:**
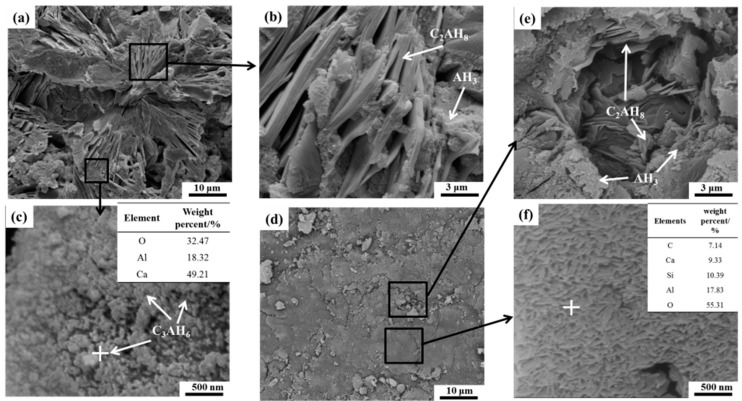
Morphologies of hydrates after hydration 24 h. (**a**–**c**): CAC paste; (**d**–**f**): CAC paste with 100 wt.% CS.

Scanning electron microscopy (SEM) was employed to study the morphology and microstructure of hydrates after 24 h hydration for CAC paste without and with 100 wt.% CS. It can be seen from Figure 3a that petal-shaped hydrates with a porous structure are formed in the CAC paste without CS. A large amount of well-developed plate-shaped C_2_AH_8_ and particles AH_3_ can be observed in Figure 3b, and AH_3_ particles are inset between the C_2_AH_8_ compounds. Some C_3_AH_6_ particles can be seen in the void in Figure 3c. However, as seen in Figure 3d, the microstructure of the CAC paste with CS is different to that of the pure CAC paste. It is compact and featureless, and a small amount of C_2_AH_8_, C_3_AH_6_ and AH_3_ compounds occur at some loose places, as seen in Figure 3e. The compact structure in Figure 3d is actually composed of a porous rod-like compound, which contains calcium silicate and aluminate, as shown by EDS in Figure 3f. The formation of vaterite (CaCO_3_) is the result of the reaction between hydrates and ambient CO_2_, indicating that the formation of crystalline hydrates also occurs owing to a dissolution/precipitation mechanism. These results are consistent with the XRD patterns in Figure 2.

The nucleation process only happens around the cement particles in the plain CAC paste, and hydrates are therefore formed around the cement particles [24], creating the petal-shaped microstructure. However, the SiO_2_ nanoparticles are dispersed not only around the cement particles, but also in the pores between the cement particles [10,18]. Therefore, due to the nucleation effect and wide distribution of the SiO_2_ nanoparticles, the hydrates are formed around the cement particles and pores, leading to the compact structure in the paste. The compact structure also restrains the growth of hydrates, leading to the small amount of hydrates in the CAC paste with CS.

### 3.2. Thermal Analysis

**Figure 4:**
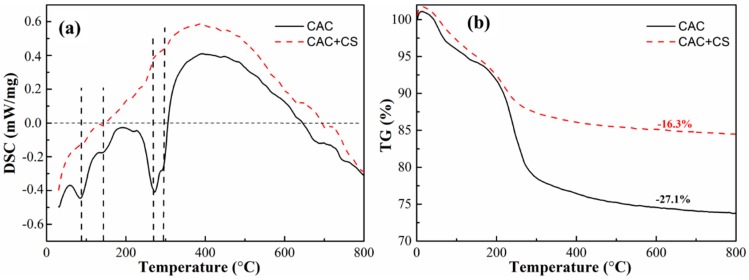
DSC-TG curves for CAC paste without and with 100 wt.% CS after hydration 24 h. (**a**) DSC; (**b**) TG.

The Thermogravimetry-Differential scanning calorimetry (DSC-TG) curves for CAC paste without and with 100 wt.% CS after 24 h hydration are presented in Figure 4. Figure 4a shows the different peaks for the two kinds of pastes. In detail, it shows four endothermic peaks in the vicinity of 100 °C, 160 °C, 260 °C and 300 °C for the CAC paste without CS. In contrast, no obvious endothermic peaks are observed in the CAC paste with CS. With increasing temperature, the hydrate transformation can be observed as follows. When the temperature is below 21 °C, the main hydrate is CAH_10_; as the temperature rises to above 21 °C but below 35 °C, CAH_10_ is transformed into C_2_AH_8_ and AH_3_. As the temperature continuously increases to 35 °C, both CAH_10_ and C_2_AH_8_ is transformed into C_3_AH_6_ and AH_3_ [24,25]. Therefore, during the heating process in the thermal analysis, the above dehydration processes are generated and their crystal water in the hydrates is lost below 100 °C. While C_2_AH_8_ decomposes at about 160 °C, AH_3_ loses its molecules in the form of water vapor at 260 °C, and C_3_AH_6_ decomposes at 300 °C by the following Equations (1)–(3).

(1) $${\mathrm{AH}}_{3}\to A+3H$$

(2) $${3C}_{2}{\mathrm{AH}}_{8}\to {2C}_{3}{\mathrm{AH}}_{6}{+\mathrm{AH}}_{3}+9H$$

(3) $${7C}_{3}{\mathrm{AH}}_{6}\to C_{12}A_{7}+9\mathrm{CH}+32H$$

However, no obvious endothermic peaks in the CAC paste with CS can be ascribed to the exothermic reaction of SiO_2_ nanoparticles during the heating process. The SiO_2_ nanoparticles are apt to agglomerate to reduce the surface energy in the heating process, releasing a lot of heat. The heat releasing capacity for the SiO_2_ nanoparticle agglomeration may be much more than the heat absorption capacity of the hydrate decomposition [22], so exothermic peaks instead of endothermic peaks can be seen in Figure 4a.

The TG curves in Figure 4b show that the weight loss of plain CAC paste is 27.1%, which is higher than that of CAC paste with CS. Because the free water in the pastes has been volatilized in the freeze drying process, the weight loss of the paste is mainly due to the dehydration of the hydrate; therefore the TG curves indicate that there are few hydrates in the CAC paste with CS. In other words, the late stage of the CAC hydration process is inhibited by CS, leading to fewer hydrates in the paste.

### 3.3. FT-IR Spectra Analysis

**Figure 5:**
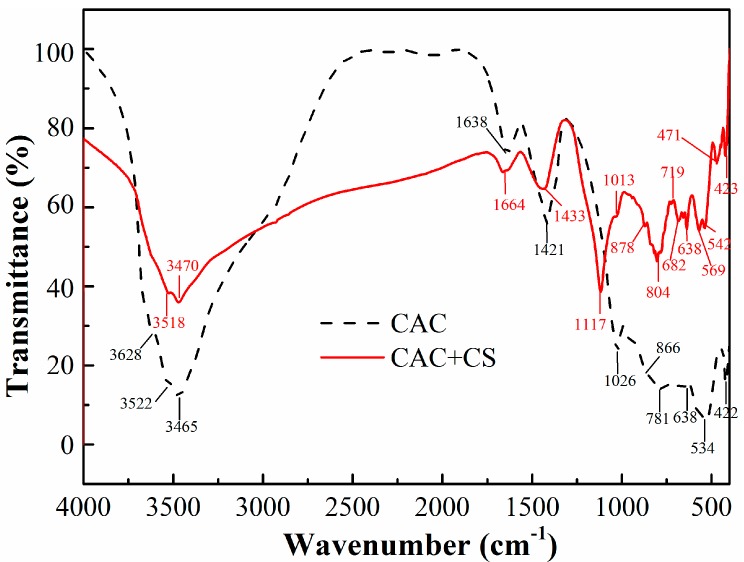
FT-IR spectra of CAC paste without and with CS.

The FT-IR spectra of CAC paste after 24 h hydration are given in Figure 5. The results show the presence of bands located at 3628–341 cm^−1^, which is assigned to the hydrates. It is obvious that the intensity of the absorption bands is decreased when CS is added. The presence of a band at 1638–1664 cm^−1^ is due to the stretching and bending vibration of the water lattice in the hydrated calcium aluminate [26]. Its intensity decreases when CS is added because the amount of hydrated calcium aluminate is decreased. The band at 1421–1433 cm^−1^ is the C-O bond stretching of CO_3_^2−^, resulting from the carbonation of hydrated aluminate. Exposure of cement pastes to air results in a very quick carbonation [27]. The sharp absorption band at 1117 cm^−1^ is the bending vibration of Si-O-Si. The bands at 1013 and 719 cm^−1^ are attributed to the bonds vibration of C_2_ASH_8_ [28]. The signals at about 680 and 640 cm^−1^ are assigned to AlO_6_ vibrations. The band at 804 cm^−1^ is related to the Si-O vibration in the amorphous SiO_2_. The band at 471 cm^−1^ is due to the bending vibration frequency of the Si-O bond of the SiO_4_^2−^ tetrahedral, and an absorption band associated with the Si-O-Al vibration lies at 542 cm^−1^, which are well defined [29]. The band observed at about 422 cm^−1^ is the vibration of C_2_AH_8_/CAH_10_.

It is noted that the intensities of the absorption bands are decreased as CS is added into the paste, because the phases of non-ordered structures could cause the band width to increase and intensity decrease. This result is in agreement with the XRD patterns seen in Figure 2. The bands at 1013 and 719 cm^−1^ confirm the formation of C_2_ASH_8_, indicating that the silica nanoparticles have reacted with CAH10/C_2_AH_8_. The stable hydrate C_2_ASH_8_ is in favor of mitigating the strength lost during hydrate conversion.

### 3.4. Electrical Conductivity Analysis

**Figure 6:**
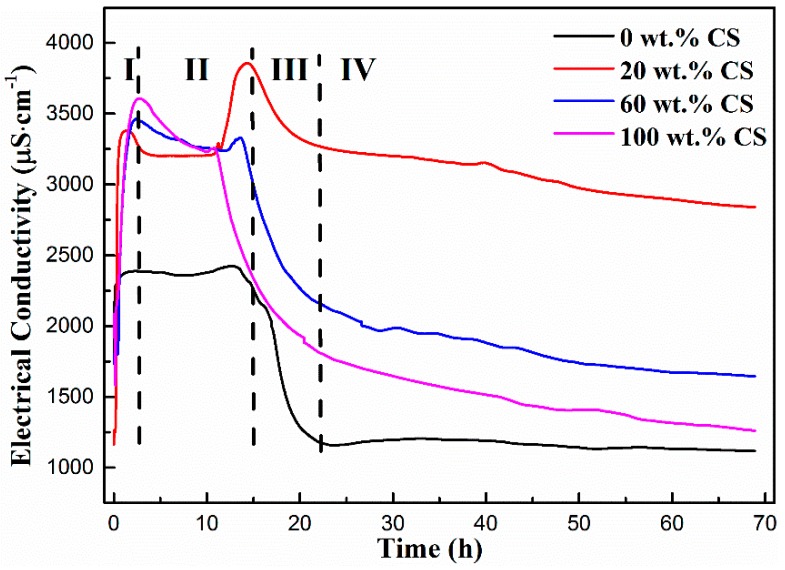
Electrical conductivity curves of pastes with contents.

Electrical conductivity can be used as an indication for setting characteristics as well as structural changes of the hardened pastes. To understand the ion concentration variations and structural changes during the hydration process, the electrical conductivity curves of pastes with different CS contents are shown in Figure 6. It is clear that there are four stages for the electrical conductivity curves. The electrical conductivities increase rapidly during the first stage I, and are then stable during the second stage II. After that, the electrical conductivities decrease at the third stage III, and finally reach their lowest value and stay stable during stage IV. The increase of electrical conductivity in the initial period of hydration is mainly due to the dissolution of CA grains according to the following Equation.

(4) $${\mathrm{CaAl}}_{2}O_{4}{+4H}_{2}O\to {\mathrm{Ca}}^{2+}{+2\mathrm{Al}(\mathrm{OH})}_{4}{}^{-}$$

In the first minute of hydration, a high concentration of Ca^2+^ and Al(OH)_4_^−^ is liberated into the paste [24,30]. Therefore, the increase of ion concentration and mobility (Ca^2+^ and Al(OH)_4_^−^) in the pastes improves the electrical conductivity during the initial hydration process. These ions are readily absorbed to form a thin layer of hydration products, which then creates an envelope around the unhydrated cement grains. This envelope consists of electrical double layers, and it absorbs calcium ions and counter ions, which decreases the increments of ion number and mobility [31] and obtains the maximum values of electrical conductivity. This period lasts almost 1 h. After that, the rate of ion dissolution and nucleation reaches a balanced state, and the electrical conductivity of the pastes is then stable at this second stage. During the third period of hydration, the formation and accumulation of various kinds of hydrates result in a remarkable consumption of ions leading to a sharp decrease in the electrical conductivity of the pastes. The hydration process is almost completed in the fourth period and the hydration compounds are formed, so the electrical conductivity is therefore stable.

It is worth noting that the maximum value of the CAC paste with CS is almost 3800 μS⋅cm^−1^, which is much higher than that of the plain CAC paste (2400 μS⋅cm^−1^). Further, the most interesting feature is that there are two peaks in the electrical conductivity curves as CS is added into the CAC paste. Therefore, it is assumed that the high electrical conductivity of the paste with CS can be attributed to the effect of SiO_2_ nanoparticles in CS. The cement dissolution keeps the Ca^2+^/Al(OH)_4_^−^ ratio in its equilibrium condition. However, the SiO_2_ nanoparticles can be coagulated in the CAC paste due to the existence of Ca^2+^, leading to the consumption of Ca^2+^ in the solution [32]. This coagulation process increases the concentration of Al(OH)_4_^−^ according to Equation (4), thus the maximum electrical conductivity value is increased. Further, the hydration process during the late stage is retarded due to the addition of CS, which will be mentioned in the following results. Therefore, the porosity is large and the pore structure connectivity is integrity, which is in favor of the transportation of Ca^2+^ and Al(OH)_4_^−^ in the pastes and increases the electrical conductivity. The Na^+^ as a stabilizer in the CS is also beneficial for increasing the mobility and concentration of ions and enhancing the electrical conductivity of the paste. The second peak of the electrical conductivity curves may be due to the osmotic pressure development around the cement grains. A gel layer is formed around the CA grains in the hydration process. On progressive hydration, the osmotic pressure of the gel layer increases gradually and breaks the gel layer, leading to ions going into the solution [33].

In addition, the proportion of added CS affects the initial electrical conductivity values and the location of the maximum value peaks, as shown in Figure 6. It can be seen that the initial electrical conductivity values are increased gradually as CS contents increase, indicating that the rate of initial hydrolysis of CAC is increased. Further, the locations of the maximum value of electrical conductivity shifting to high position can be attributed to the accelerating effect of CS during the early stage of the hydration process.

### 3.5. Hydration Heat and Kinetic Model Analysis

**Figure 7:**
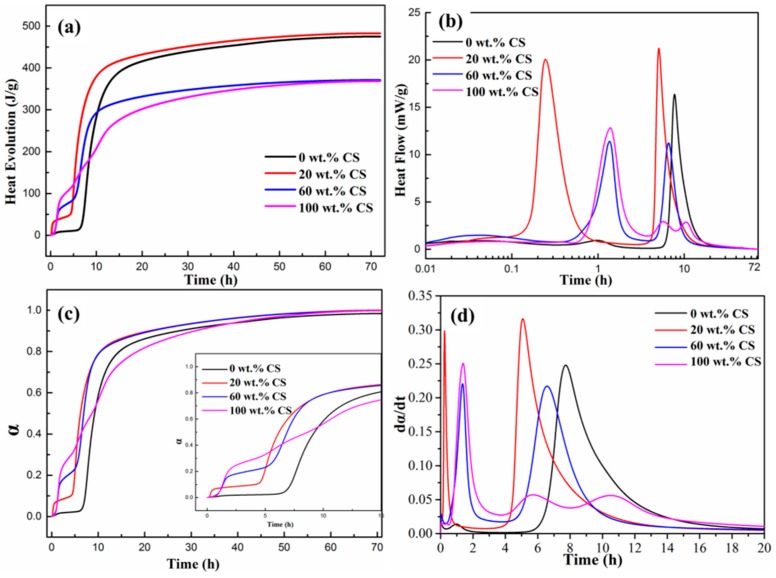
Hydration heat curves of CAC without and with CS. (**a**) Heat evolution curves; (**b**) heat flow curves; (**c**) hydration degree curves; (**d**) hydration heat rate curves.

The hydration heat of cement pastes in isothermal conditions is used as an indicator of the advancement of hydration reactions. The heat evolution and heat flow curves of cement pastes without and with CS are shown in Figure 7. It can be seen from Figure 7a that the hydration heat of plain CAC paste is 475.16 J/g, which is almost equal to the hydration heat of the paste with 20 wt.% CS (483.07 J/g, joules per gram of cement). However, as the CS contents are increased to 60 wt.% and 100 wt.%, the hydration heat capacities are decreased to 371.94 J/g and 368.99 J/g, respectively. The lower hydration heat of the paste can be attributed to the hydration degree decreasing of CAC. Owing to the nucleus of SiO_2_ nanoparticles around the cement particles, the hydrates covered with cements are dense during late stage of the hydration process. As more CS is added, the hydrates covered with cement particles are denser during the late stage (including the deceleration and stable periods) of the hydration process, which inhibits the water molecular and ion transportation between the unhydrated cement and paste solution. Another probability is that a considerable amount of free water is constrained in the pores of silica nanoparticle aggregates during the agglomeration process, resulting in the reduction of water for hydration of the cement [34]. Further, the formation of C_2_ASH_8_ also leads to the reduction of water in the paste, decreasing the hydration degree of CAC [18]. Therefore, the heat releasing content of hydration is decreased as CS is added.

The heat flow curves in Figure 7b show that there are two peaks for all samples. The two exothermic peaks are located at 1 h and 10 h for plain CAC, respectively. However, the intensities of the two exothermic peaks for the pastes with CS are higher, while the positions of corresponding peaks are lower than those of the plain CAC paste, indicating that the hydration exothermic rate for pastes with CS hydrates is high. The cement hydration process is a heat releasing process, which usually contains two peaks. The first peak is relevant with the wetting heat, dissolution saturation and nucleus formation of cement as the cement is in contact with water; the second is due to the precipitation of the hydrates [17,35]. When CAC cement is in contact with water and CS, Ca^2+^,OH^−^,Al(OH)_4_^−^ ions are produced, and Na^+^, SiO_3_^2−^ are also presents in the CS. The SiO_2_ nanoparticles begin to play the role of a nucleus and consume the Ca^2+^, making the Equation (1) moving to the right side. What’s more, the silica nanoparticles shorten the induction period of the cement paste through reducing the concentration of calcium ions [27,32]. Therefore, the first heat releasing peak appears earlier in the paste with CS.

The degree of hydration, defined as the relative amount of hydrated cement, is often measured experimentally by an isothermal calorimeter. The degree of hydration α(t) can be computed as a function of the heat release *Q*(t) and the heat release when complete hydration is reached *Q*_∞_[17], as shown in Equation (5).

(5) $$\alpha \left(t\right)=\frac{Q\left(t\right)}{Q_{\infty }}$$

The hydration degree curves with CS contents are shown in Figure 7c. It can be seen that the induction period of plain CAC paste is almost 7 h, much longer than that of pastes with CS (less than 2 h). The hydration rate curves in Figure 7d obviously show that the hydration rate is greatly increased with addition of CS, especially for the first heat releasing process, suggesting that the hydration rate at the early stage of the hydration process is greatly prompted by silica nanoparticles.

The Krustulovic-Dabic kinetic model assumes that there are three basic hydration processes; nucleation and crystal growth (NG), interactions at the phase boundaries (I) and diffusion (D). It is suggested that these three processes may take place simultaneously, but the whole hydration process is controlled by the slowest one. NG dominates the initial stage of hydration, and, as the reaction continues, I or D replace NG as the dominant process. The following Krustulovic-Dabic Equations and the corresponding logarithmic Equations (6a)–(8b) are usually applied to describe the hydration kinetics [36,37]. 

NG process:

(6a) $${\left[-\ln\left(1-\alpha \right)\right]}^{1/n}{=K}_{\mathrm{NG}}t$$

(6b) $$\ln\left[-\ln\left(1-\alpha \right)\right]{=\mathrm{nlnK}}_{\mathrm{NG}}+\mathrm{nln}\left({t-t}_{0}\right)$$

I process:

(7a) $$1-{\left(1-\alpha \right)}^{1/3}{=K}_{I}t$$

(7b) $$\ln\left[1-{\left(1-\alpha \right)}^{1/3}\right]{=\mathrm{lnK}}_{I}+\ln\left({t-t}_{0}\right)$$

D process:

(8a) $${\left[1-{\left(1-\alpha \right)}^{1/3}\right]}^{2}{=K}_{D}t$$

(8b) $$2\ln\left[1-{\left(1-\alpha \right)}^{1/3}\right]{=\mathrm{lnK}}_{D}+\ln\left({t-t}_{0}\right)$$

where n is the value of the Avrami exponent that relflects the nucleation and growth mechanism details. K_NG_, K_I_ and K_D_ are the reaction rate of the NG, I and D processes, respectively. 

**Figure 8:**
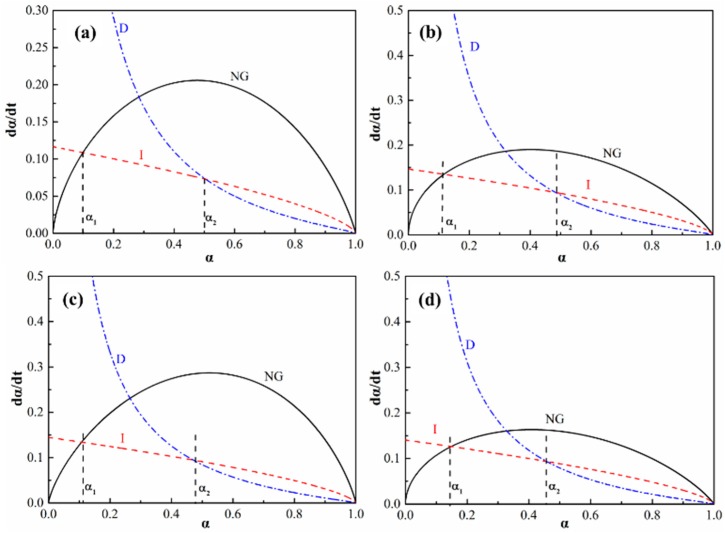
The hydration rate-hydration degree of cement pastes. (**a**): 0 wt.% CS; (**b**): 20 wt.% CS; (**c**): 60 wt.% CS; (**d**): 100 wt.% CS.

Using the data of α and t listed in Figure 7a,b, and based on the Equations (6b)–(8b), the curves of dα/dt versus α can be determined and are shown in Figure 8. The α_1_ and α_2_ in the curves represent the transition points from the NG to I process and from the I to D process, respectively, which are listed in Table 3. The curves in Figure 7 suggest that the initial stage of hydration is dominated by the NG process because there is sufficient water and less hydrates at this stage. As the hydration reaction continues, the amount of hydrates increases resulting in difficult migration environment for ions. Thus, the hydration process is transformed into the diffusion-dominated stage (I), and lastly the diffusion stage (D) controls the hydration rate. However, there is a slight difference in the effect of CS on controlling the hydration rate of cement, as shown in Table 3. It is shown that the first transition point of α_1_ increases with increasing CS content, while the second transition point of α_2_ decreases gradually. The α_1_ and α_2_ of pure cement paste are 0.1 and 0.5, respectively. When 100% CS is added, the α_1_ and α_2_ vary to 0.14 and 0.46, respectively. The increase in α_1_ indicates that the SiO_2_ nanoparticles promote the hydration rate in the NG process period, and the extension of the NG process suggests that the SiO_2_ nanoparticles develop well as hydrate nuclei. While the decrease in α_2_reveals that the phase boundaries stage (I) is shortened, which the hydration reaction transformed into diffusion stage (D) earlier occurs. This transformation of the dominant reaction is due to the nucleus role of SiO_2_ nanoparticles. As can be seen in Figure 3c,d, the microstructure of the paste with CS is dense, which inhibits the water molecule and ion immigration, so the dominant hydration reaction earilytransforms into diffusion stage. Thus, the SiO_2_ nanoparticles promote the hydration degree during the NG process stage and decrease the hydration degree at the phase boundaries stage, and therefore the hydration reaction earlier transforms into the diffusion stage.

### 3.6. ^29^Si MAS NMR Spectroscopy

**Figure 9:**
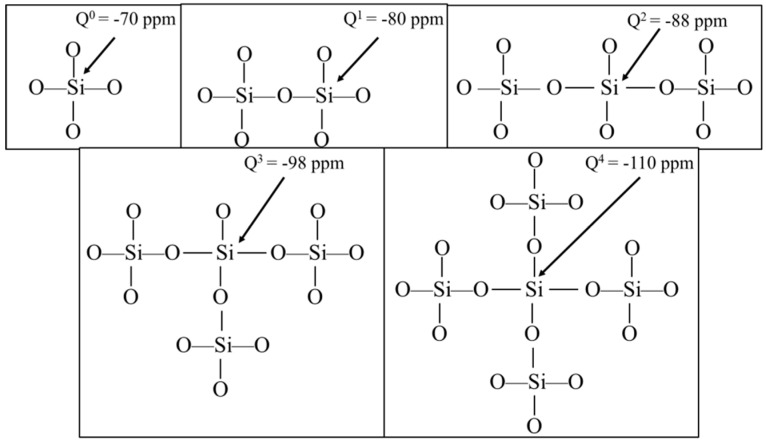
Various arrangements of silicon–oxygen bonding.

Solid-state nuclear magnetic resonance spectroscopy (NMR) is one of the most powerful tools for studying the reactions of cement hydration. Various forms of Si-O bonding are shown in Figure 9. The basic tetrahedral unit, (SiO_4_)^4−^, is referred to in this field as a Q^0^ unit, where the superscript on the Q refers to the number of (SiO_4_)^4−^ attached to the central (SiO_4_)^4−^ unit. Q^1^ refers to a dimer and Q^2^ represents silicon atoms within a polymeric chain of (SiO_4_)^4−^ units. Q^3^ and Q^4^ correspond to silicon atoms from which increasingly complex degrees of chain branching occur.

**Figure 10:**
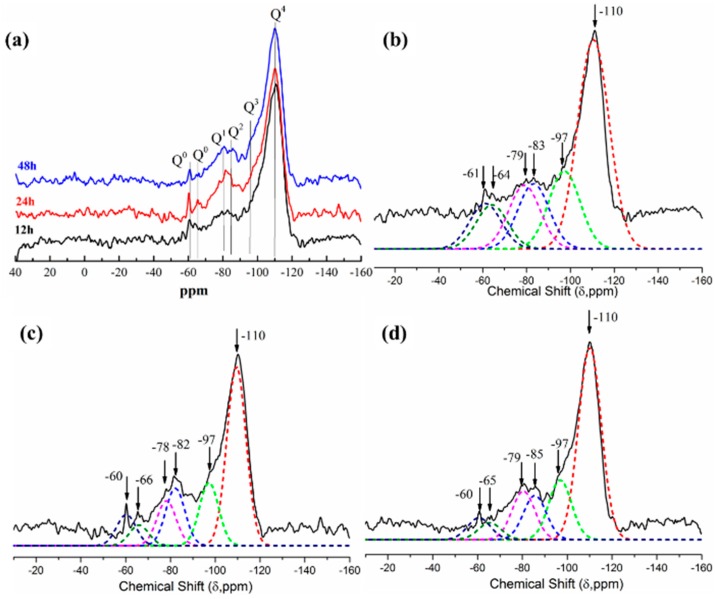
^29^Si spectrum of CAC paste with 100 wt.% CS after hydrating different hours. (**a**) The overall spectra; (**b**) 12 h; (**c**) 24 h; (**d**) 48 h.

The local environments of Si atoms in hydrated pastes are determined by ^29^Si MAS NMR spectroscopy, as shown in Figure 10. The spectra in all the samples analyzed are formed by six peaks at ~−110, −97, ~−82, ~78 ppm and two peaks at ~60 and 65 ppm. The partial dissolution of the silica particles produced depolymerized species that could interact with Ca and Al ions. The ^29^Si spectrum of the amorphous silica displays a single peak at −110 ppm, which is associated with tetrahedral Si in Q^4^[Si(OSi)_4_] environments (Figure 9). The intensity of the −110 ppm peak is important in the hydrated compounds, as it indicates that a considerable part of the silica has not reacted. The peak at approximately −97 ppm corresponds to Q^3^[Si(OSi)_3_OH] environments at the surface of the silica nanoparticles [38,39]. The amount of unreacted silica is estimated from the relative intensity of the −110 and −97 ppm peaks, which are associated with Si atoms in Q^4^ and Q^3^ environments. The formation of [SiO_4_]_4_ groups in the hydrogarnet is ascribed by the presence of the ^29^Si NMR peak at ~−60 and −65 ppm. These signals correspond to isolated [SiO_4_] groups in Q^0^(4Al) environments. It can be seen from Figure 10 that the intensities of Q^0^ peaks at ~−60 and −65 ppm are increased with hydration time extension, suggesting that the amount hydrates in hydration reactions increases, which is consistent with the FT-IR spectra results that show Si-O-Al vibration in the hydrates.

### 3.7. Hydration Mechanism of CAC

**Figure 11:**
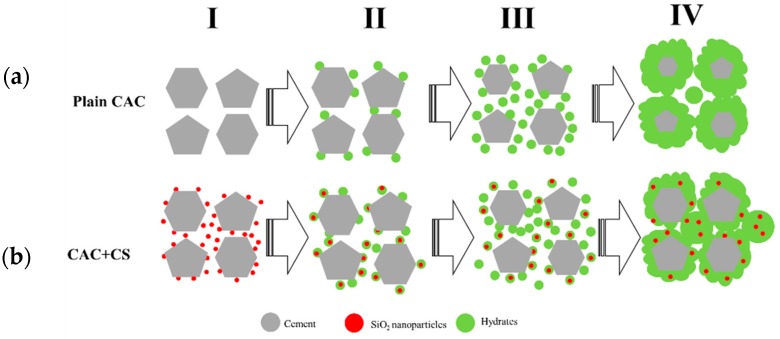
Schematic diagram of the hydration process of cement without and with CS. (**a**) Plain CAC; (**b**) CAC with CS.

Figure 11 displays the schematic diagram of cement hydration processes according to the above results and analysis. For the cement without CS the hydration process is composed of four periods; the induction period, acceleration period, deceleration period and stable period [17,24]. As can be seen in Figure 11a, after the dissolution of cement in water, the crystal nucleus of hydrates is formed at the surface of the cement during the induction period. Next, the hydrates are precipitated during the acceleration period. Owing to hydrate coverage at the surface of the cement during the hydration process, the dissolution process of cement is inhibited in the deceleration period. When the cement particles are covered with dense hydrates and the water is consumed, the hydrate process is dominated by diffusion and the stable period is initiated.

However, the cement particles are surrounded with silica nanoparticles when they are mixed with CS, as shown in Figure 11b. These silica nanoparticles are the nucleus point for hydrates, so the hydration process are conducted fast to acceleration period [1,4,10], therefore the hydration heat and electrical conductivity of cement with CS are higher than those of cement without CS during the early stage of the hydration process, as shown in Figure 6 and Figure 7. AS the hydration proceeds, the cement particles are covered with dense hydrates, as shown in Figure 3, so the dominant hydration reaction is transformed into the diffusion stage earlier as shown in Figure 8. 

The incorporation of nanoparticles in CAC cement was investigated in previous studies, which showed that nanoparticles accelerate the cement hydration due to the nucleus effect. However, in light of all the results in this work, when hydrated, SiO_2_ nanoparticles only accelerate the early stage of hydration, but inhibit the late stage of hydration for CAC cement. Owing to the nucleus effect of SiO_2_ nanoparticles, a gel layer forms around CAC grains during the early stage of hydration [33]. The gel layer grows dense as hydration continues, and inhibits the transportation of water and ions between the solution and cement grains, resulting in the hydration degree reducing. Because of this, the hydration degree at the NG (nucleation and crystal growth) process stage is promoted, while it is limited at the phase boundaries stage, and the diffusion stage in the hydration reaction is brought forward. This suggests that the nanoparticles with high specific area facilitate an effective role of obtaining a cement paste with a highly rapid coagulation rate.

## 4. Conclusions

In this work, the effects of colloidal silica on the hydration process of cement were investigated. The phases and microstructure evolution were characterized, and the electrical conductivity and hydration heat were measured, NMR and kinetic models were analyzed to discuss the influence mechanism of colloidal silica on the CAC hydration process. The following are the key aspects.

(1) Owing to the nucleation effect and wide distribution of SiO_2_ nanoparticles, a dense structure was formed in the CAC paste with CS, while petal-shaped hydrates with a porous structure were formed in the plain CAC paste. The differences in the microstructure suggest that the hydration reaction occurs not only around the cement grains, but also in the pores between cement grains.
(2) For the hydration process of CAC paste, the silica nanoparticles acting as a nucleus promoted the early stage of hydration, but inhibited it during late stage (including the deceleration and stable periods). Because of this, the maximum electrical conductivity value for the CAC paste with CS was higher than that of the plain CAC paste, while the hydration heat of the CAC paste with CS was lower.
(3) The dominant reaction was transformed into the diffusion stage earlier in the CAC paste with CS, resulting in the different roles of SiO_2_ nanoparticles in the hydration process. The SiO_2_ nanoparticles promoted the hydration degree during the NG process stage and decreased the hydration degree at the phase boundaries stage.

Therefore, it is necessary to add some retarder to slow down the early hydration of CS and CAC paste, because the SiO_2_ nanoparticles promote the early stage of hydration to shorten the setting time. And there needs longer curing time for CS and CAC as binder to fully hydrate, as the hydration process earlier enter into diffuse stage and inhibit it during late stage of hydration. In conclusion, in order to obtain enough strength of castables, the curing time for CS and CAC as binder will be longer than that of plain CAC as binder.

## Figures and Tables

**Table 1:** Compositions of calcium aluminate cement (CAC) and colloidal silica (CS) (wt.%).

Compositions	Al_2_O_3_	CaO	SiO_2_	Fe_2_O_3_	Na_2_O
CAC	70	29	0.15	0.05	0.25
CS	0.358	0.065	30−31	—	2.63

**Table 2:** Proportions of CAC and CS (wt.%).

Compositions	Proportion			
CAC	100	100	100	100
CS	0	20	60	100

**Table 3:** The transition points between NG, I and D processes.

Sample	α_1_	α_2_
0%	0.1	0.5
20%	0.12	0.49
60%	0.13	0.48
100%	0.14	0.46
